# Matching Multiple Backgrounds: Egg Camouflage Across Different Habitats in a Shorebird

**DOI:** 10.1002/ece3.72847

**Published:** 2026-01-09

**Authors:** Alexandra Grandón‐Ojeda, Tamás Székely, Robert N. Kelsh, Alejandro Pérez‐Hurtado, Innes C. Cuthill

**Affiliations:** ^1^ School of Biological Sciences University of Bristol Bristol UK; ^2^ Escuela de Medicina Veterinaria, Facultad de Recursos Naturales y Medicina Veterinaria Universidad Santo Tomás Santiago Chile; ^3^ Department of Life Sciences and Milner Centre for Evolution University of Bath Bath UK; ^4^ HUN‐REN‐DE Reproductive Strategies Research Group, Department of Evolutionary Zoology and Human Biology University of Debrecen Debrecen Hungary; ^5^ Departamento de Biología Universidad de Cádiz Cádiz Spain

**Keywords:** animal camouflage, background matching, egg coloration, nesting behaviour, protective coloration

## Abstract

For species found in multiple habitats, the problem of camouflage against visually different backgrounds can be challenging. This is particularly so for bird eggs in open nests, as the scope for movement or alternative defensive strategies is limited. We studied egg camouflage in a small shorebird, the Kentish plover *Anarhynchus alexandrinus*, in two different coastal habitats in Cádiz province, Spain: sandy beaches and saltmarshes. Using calibrated photographs taken in situ and neurophysiologically plausible models of colour and pattern vision, we assessed the predicted discriminability of egg colour and patterning from those of backgrounds for likely nest predators (avian and mammalian carnivore) and, for comparison, humans. The findings suggest that at close range *A. alexandrinus* eggs are more susceptible to detection by visual predators based on their patterns (aka visual texture) rather than their colours, but at distances beyond which individual pattern elements can be resolved, they are highly cryptic. Although the colours and patterns of the saltmarsh and beach nest sites differ, the colours and surface patterning of eggs do not, suggesting that there is no local adaptation. However, the colours of eggs are similar to the types of background colours that overlap between the beach and saltmarsh. This suggests that, although the gross visual appearances of beach and salt marsh are quite different, egg camouflage in Kentish plovers relies on behavioural nest‐site selection and a good colour match to the average location type. The maculation on the eggs does not appear to represent background matching in terms of pattern, so its function remains speculative.

## Graphical abstract

We studied egg camouflage in Kentish plovers nesting in two visually distinct habitats, sandy beaches and salt marshes, in southern Spain. Despite initial expectations of local adaptation or compromise camouflage, the birds instead chose nest sites within each habitat that shared overlapping colour and visual texture characteristics, allowing effective camouflage from predators, especially at a distance. Our study exemplifies the importance of determining the confusability (misclassification) of whole distributions of prey and background features, not just the differences in the means of those distributions or the perceptual differences between particular colours.

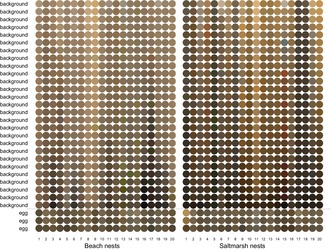

## 1 Introduction

In ground‐nesting birds, nest predation is often the primary factor contributing to reproductive failure (Fulton 2019; MacDonald and Bolton 2008; Martin 1995; Martin and Li 1992; Menezes and Marini 2017). Costs can be reduced by behavioural strategies such as covering the nest with vegetation or sand (Gómez, Ramo, Troscianko, et al. 2018; Summers and Hockey 1980; Troscianko, Wilson‐Aggarwal, Spottiswoode, and Stevens 2016) or the distraction from, or physical defence of, nests (Gómez‐Serrano and López‐López 2017; Walters 1982). However, because eggs cannot themselves move and parents are not always present, camouflaged shells remain a likely primary adaptation against predation (Kilner 2006; Troscianko, Wilson‐Aggarwal, Stevens, and Spottiswoode 2016; Westmoreland and Kiltie 1996), even though other selective forces influence egg colour (Hanley et al. 2013; Kilner 2006; Stoddard et al. 2011; Wisocki et al. 2020). In particular, among the Plovers (Charadriinae), many species' eggs have been described as exhibiting colouring and patterning that mimic their surroundings, such as rocks, vegetation or sand (Greeshma and Jayson 2018; Kaur and Khera 2017). Furthermore, Skrade and Dinsmore (2013) found that the degree of colour contrast between mountain plover ( *Charadrius montanus* ) eggs and their nest surroundings predicted the likelihood of successful completion of incubation and subsequent survival to hatching. However, these and most other studies assessing egg camouflage have used human judgements or human‐specific colour measures, so the extent to which eggs are actually cryptic in the eyes of their predators remains under‐researched (Stoddard et al. 2011, 2016; Troscianko, Wilson‐Aggarwal, Spottiswoode, and Stevens 2016; Troscianko, Wilson‐Aggarwal, Stevens, and Spottiswoode 2016).

Effective camouflage is often determined by behaviour in addition to surface pigmentation (Stevens and Ruxton 2019). For example, both Stevens et al. (2017) and Gómez, Ramo, Troscianko, et al. (2018) showed that individual plovers (and some other ground‐nesting species) not only selected nesting locations to which their eggs were closely matched in predator‐specific colour measures, but also changed the visual environment by covering their nests with materials that blended in with the surroundings. Hancock et al. (2023) used 3D reconstructions of the terrain around Northern Lapwing ( *Vanellus vanellus* ) nests to show that, despite nesting in the open, the parents exploited slight variations in elevation such that the eggs would not be visible to a terrestrial predator from even 1.5 m away. In addition to this, whether assessed using visual modelling of fox or raptor vision, the eggs were an excellent colour match to backgrounds of fallow or bare fields, but less so vegetated areas. That parents might be aware of their individual degree of camouflage is also suggested by a study by Wilson‐Aggarwal et al. (2016). When a simulated predatory threat approached the nest (a human), ground‐nesting plovers fled, but those with poorer camouflage (as assessed by visual modelling) flew off when the ‘predator’ was further from the nest. This suggests that the birds were aware that the poorer their camouflage, the greater their risk of detection. In terms of direct evidence for background choice with respect to egg colour, Lovell et al. (2013), conducted a study in which female quail, *Coturnix japonica* , exhibited a preference for laying their eggs on substrates that reduced detectability specifically for their own eggs' patterning. Quail with moderate‐to‐high levels of maculation (speckling) on their eggs chose substrates that matched the dark maculation, while quail with eggs having the least maculation chose lighter backgrounds that matched the ground colour of their eggs. Alothyqi et al. (2024) further experimentally demonstrated that their substrate choice for egg‐laying changed with breeding experience, such that the colour match was improved. Interestingly, there was no difference between naïve and experienced breeders in their preference for substrates that enhanced disruptive camouflage. This suggests that quails have a preference for laying on substrata that align with the average coloration of their eggs, and that this is learnt. By inference, this would minimise the probability of being detected by visual predators.

In the present study, we use visual modelling to assess how easily Kentish plover (*Anarhynchus alexandrinus*) eggs can be discriminated from their backgrounds in two visually different types of habitat in southern Spain: sandy beaches and salt marshes. Subjectively, these two habitat types differ in terms of the background against which eggs would be viewed by a predator, so first, we quantify the visual differences and then we investigate whether there is evidence of local adaptation (camouflage‐enhancing differences in coloration of the eggs found in the two habitats). While local adaptation might seem very unlikely because of the mobility of the study organism and proximity of the two breeding areas, we nevertheless include it as a logical possibility that can be tested. We also determine whether, if there is no difference in egg coloration between habitats, the eggs are specialised to match one habitat better or instead show ‘compromise camouflage’ (Merilaita et al. 2001; Houston et al. 2007). The latter is where a camouflage pattern is intermediate between two background types and yet, while suboptimal on either alone, has higher fitness on average across both (Hughes et al. 2019; Merilaita et al. 1999).

## 2 Methods

### 2.1 Study Site

The fieldwork was carried out in the Parque Natural de la Bahía de Cádiz, near Cádiz in south‐west Spain. Nests were located with the help of local ornithologists who were ringing plovers and monitoring reproduction as part of a long‐term conservation programme (ringing permits to APH from SEO/Birdlife—the Spanish Ornithological Society—and the Junta de Andalucia, ref. GB‐10/22/EA/FA/AN/FSA). In a field study of camouflage, there is always the risk of sampling bias in either or both of two opposite directions: some nests may not be found by virtue of their excellent camouflage, or the poorest‐camouflaged nests have already been predated before the survey. If there is a bias in our sample, we feel the latter is more likely because, given that our research piggy‐backed on long‐term monitoring of breeding performance, the search for nests was exhaustive and the local monitoring team was highly familiar with the study sites.

All nests were photographed using a Nikon D3500 digital SLR camera with Nikon AF‐S DX 35 mm f/1.8G lens (Nikon Corp., Tokyo, Japan) and the images saved in NEF format (RAW: uncompressed, unprocessed sensor data). All photographs were taken at approximately the same distance above each nest (ca. 80 cm, waist‐height; see later for scaling adjustments), under clear skies with bright sunlight at ISO 100 at f11, with the integration time (shutter speed) varied for correct exposure. The response function of the camera with respect to spectral radiance, and the spectral sensitivity of the R, G and B sensors, had previously been determined in an optical laboratory using the methods described by Stevens and Cuthill (2006), Stevens et al. (2007) and Lovell et al. (2005). In brief, we photographed the six grey squares of an X‐Rite ColorChecker Passport (X‐Rite, Grand Rapids, MI, USA) illuminated by a voltage‐stabilised tungsten‐halogen light source under the range of camera apertures we expected to use in the field (f8 to f16, at ISO 100). The radiance of the light reflected from the same grey squares was simultaneously measured with a telespectroradiometer (TopCon, Model SR1, calibrated by the National Physical Laboratory, UK). This allowed us to determine the relationship between the light input (radiance) and output (the RGB pixel values in uncompressed RAW images). As expected (Troscianko and Stevens 2015), this was linear. The linearity of changes in camera integration time (e.g., does halving the integration time halve the captured radiance?) was also confirmed by photographing the same chart through a set of neutral density filters. Separately, we determined the spectral sensitivity of the camera's RGB sensors by photographing a white reflectance standard (Spectralon 99%; Labsphere, North Sutton, NH, USA) through a series of 31, 10 nm bandpass filters (Ealing Electronics, Watford, UK) from 400 to 700 nm, measuring spectral radiance with the telespectroradiometer at the same time. Photography and radiance measurement were done in both an upward and downward series through the wavebands, and the means used.

Knowing the response of the camera to the incoming light is not enough, because that light is a produce of both the reflectance of the objects (the values of interest) and the spectrum of the illuminating light. For that reason, a colour chart was included in all photographs (see below) to allow calibration of each image: transforming the obtained, linear, RGB values to the known values of the X‐Rite colour squares in standardised sRGB colour space (this also achieved white‐point balancing, or the equalisation of R, G and B values for grey squares). The final stage was transforming the sRGB values to appropriate species‐specific cone‐capture values. This involved calculating the camera's (in terms of standardised RGB values) and visual systems' (in terms of cone‐captures) responses to a wide range of natural reflectance spectra. Knowing the camera's RGB sensor spectral sensitivities and the cone sensitivities of our chosen animal visual systems (see below), we calculated the cone (avian, ferret and human) and camera sRGB responses to these spectra by summing the multiplied spectra, the cone/camera sensitivities and a D65 irradiance spectrum (open‐field daylight representing the field photography conditions). We then used matrix algebra to solve the quadratic equations that mapped the camera RGB values to predicted cone responses. Out of interest and as a check on the robustness of our protocols, we also tried two other methods on a subsample of photographs. First, for the determination of camera responses and mapping functions, Pike's (2011) implementation of Finlayson et al.'s (1998) method using the Macbeth 24‐colour chart squares, whose reflectance spectra can be downloaded. Pike's method, which is much less time‐consuming than ours, produced very similar mapping functions. Second, we used the ImageJ‐based Multispectral Image Calibration and Analysis Toolbox (micaToolbox 2.3; Troscianko and Stevens 2015; van den Berg et al. 2020) to both calibrate images and convert them to cone‐capture values. We obtained very similar results and recommend the micaToolbox to researchers wishing to do similar research to ours, because of its simplicity and integrated functions.

**FIGURE 1:**
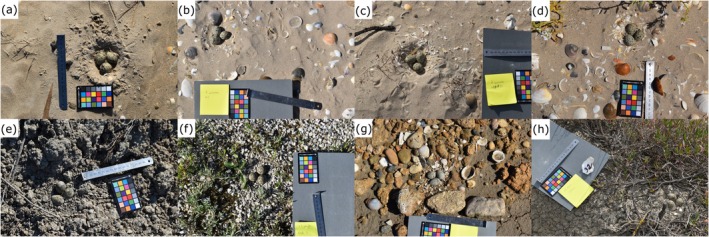
Examples of Kentish plover nests on (a)–(d) beach and (e)–(h) salt marsh in Spain, showing the type of image employed in the research. The four important elements are: the nest containing three eggs, the surrounding background, the ruler (for scale) and the colour chart (for colour calibration).

A total of 28 nests were photographed on beaches (latitude N 36°11′12.266″, longitude W 5°55′2.391″ and latitude N 36°31′29.941″ longitude W 6°13′40.895″), while an additional 60 nests were found in salt marshes (latitude N 36°30′47.029″, longitude W 6°9′4.755″), from April 15 to May 31, 2022. These two habitats were chosen because of the difference in type of background against which eggs might be viewed. Nests on beaches were often laid on fine yellow sand, with background components including shells, stones and occasional (mainly dead) vegetation or washed‐up debris (Figure 1a–d). The salt marshes were more variable, with nests found on both sand and dried mud that varied in colour from brown to grey, or on shingle (small stones), between larger patches of salt‐tolerant plants such as *Salicornia* spp. and a greater size range of stones and debris (Figure 1e–h). Nests were simple unlined scrapes (shallow depressions), and all contained three eggs at the time of surveying.

### 2.2 Photography and Image Calibration

To perform the analysis, three steps were necessary: (i) calibrating the photographs such that they represented standardised measures of reflectance rather than camera‐ and illumination‐dependent RGB pixel values; (ii) scaling the photographs to a standard size, so that the same number of pixels represented the same number of mm in every image; (iii) extraction of measures of colour and pattern relevant to different predator visual systems (Renoult et al. 2017; Stevens et al. 2007). This was possible because all photographs contained a colour standard (Colour Card 24; greywhitebalancecolourcard.co.uk) and a ruler. The colour chart had been cross‐calibrated with an X‐Rite ColorChecker Passport and was used in preference to the latter because, although probably manufactured to a lower precision, the Colour Card 24 was waterproof. Scaling was essential for comparability of the visual texture information across images because, although photographs were taken at approximately the same distance above each nest (ca. 80 cm, waist‐height), there was a small amount of variation (±5%). We scaled photographs to the resolution of the lowest‐resolution image (i.e., taken from furthest away) so that no upscaling was involved. The resolution was 12 pixels per mm, easily sufficient to capture the finer patterns of maculation on the egg. Scaling was by nearest‐neighbour interpolation to preserve the sharp dark–light edges apparent in the maculation. As a check, the process was repeated on a sample of photographs that required the greatest change from both habitats, using first bilinear and second bicubic interpolation. The change in average pixel values and the log‐Gabor filter outputs from the scaled images (i.e., the numbers used in the analyses) was < 2%.

So that the analysis program could identify the relevant objects in the pictures, it was necessary to first create ‘masks’ manually, by selecting certain areas of the photos with digital drawing tools and a mouse. A ‘mask’ is a digital layer, equal in size to the photograph, that contains 1's where the object is present and 0's where it is not. This provides a way, in software, to extract different parts of the photograph as needed. For this process, an efficient approach was to create five masks (grey standard, ruler, each of the three eggs) in the one image, each mask being of a different colour that could, subsequently, be used as a key to extract a different part of the matching photograph of a nest: three colours were used for the three different eggs (cyan, magenta and yellow), one green mask for a 40 mm length of the ruler (slightly longer than the length of a Kentish plover egg, ca. 30–36 mm, and easy to select accurately in an image) and one red mask for the third darkest grey square of the colour chart (20% reflectance). The open‐source GIMP‐2.1O photo‐editing program (www.gimp.org) was used for this, also for equivalent selection of an area for taking background samples. Then, using the grey standard and ruler masks, the photos were standardised for colour and size based on the RGB values of the grey card (of which the correct values in sRGB colour space are known) and ruler, respectively. So that samples of equivalent size and shape were used to characterise the background as for the eggs, for each photograph, one egg was randomly selected and then used as a mask to select a series of egg‐shaped samples of the background from within the background (excluding nest, ruler and colour chart). The xy coordinates for each egg‐shaped sample were selected at random, using the R function runif, but the orientation of the sample was the same as that of the egg. Twenty‐seven random, egg‐shaped background samples were taken from each photograph, such that each photograph gave 30 samples: three eggs and 27 from the background. The number 27 is somewhat arbitrary: large enough to get a better characterisation of each background's colour and pattern variation but making the calculation of the probability of correctly classifying a sample as ‘egg’ at random straightforward (3 in 30, or 0.1). Finally, the size‐and‐colour‐calibrated photos were analysed to extract the colours and pattern of eggs and backgrounds for different visual systems, using mapping functions (matrix multiplication) previously determined when the camera was calibrated. The chosen visual systems were avian, as birds of prey, gulls and corvids are the most likely visual predators, and mammalian carnivore (e.g., canids such as red fox *Vulpes vulpes* or domestic dog *Canis domesticus* and mustelids). Given the type of birds involved, arguments could be made for either a VS (violet‐sensitive, seen in birds of prey and corvids) or UVS (ultraviolet‐sensitive, seen in gulls) visual system being relevant for avian predators (Hunt et al. 2009; Hunt et al. 2007). However, for the objects and backgrounds in our study, most of the variation is in the medium‐to‐longwave, where cone sensitivities of the VS and UVS species are similar. We used the cone sensitivities of the peafowl *Pavo cristatus* from Hart (2002), this being the most widely used model of an avian VS visual system; for example, as implemented in micaToolbox (Troscianko and Stevens 2015) and pavo (Maia et al. 2019). It is also relevant to consider the visual system of the plovers themselves if there is nest‐site selection based on background colour and pattern. Based on the fact that the related golden plover ( *Pluvialis apricaria* ) has VS cones (Ödeen et al. 2009) and a lens and cornea that have a wavelength of 50% absorption of ca. 380 nm (Olsson et al. 2021), their UV sensitivity is probably not that much greater than that of humans. Mammalian carnivores are dichromats and the visual system used was that of the ferret *Mustela furo* (Calderone and Jacobs 2003). Colours were also modelled for human vision, purely for comparison and to provide a form of ground‐truth (did the model outputs match what we could see in the photographs?). All calibration and extraction of colour and pattern measures were done using a custom program, written by I. Cuthill, in R (R‐Core‐Team 2020) and the package OpenImageR (Mouselimis 2023).

### 2.3 Colour Analysis

The colour and pattern analysis followed that in Michalis et al. (2017) and Barnett, Cuthill, and Scott‐Samuel (2018) and Barnett, Michalis, et al. (2018). ‘Colour’ was defined by three variables: luminance (achromatic brightness), red‐green chromatic contrast and yellow‐blue chromatic contrast. For humans, the L*a*b* colour space is a well‐characterised representation of this type (L = luminance, a = red‐green, b = yellow‐blue) in which distances in this three‐dimensional colour space match perceived colour differences (CIE 1976). An avian equivalent was also calculated, where luminance was represented by the calculated photon catches of the double‐cone receptors, red‐green by the contrast in photon catches of the long‐ and medium‐wave single cones and yellow‐blue by the contrast in photon catches of the long‐ and medium‐wave single cones combined compared to the short‐wave cones (see discussion and justification in Xiao and Cuthill 2016). While we go on to analyse differences in these red‐green and yellow‐blue opponent measures, it is important to remember that the eggs, and to a large extent the backgrounds, are all shades of brown. The UVS or VS cone was not used in calculations because, without a camera with distinct blue and violet (or ultraviolet) sensors, the values for UVS/VS and SWS cones are very highly correlated. That UV information can be ignored is an untested assumption and, although likely low, UV reflection of Kentish plover eggs is non‐zero (back‐transforming the supplementary data on log‐UV‐reflectance in Gómez, Ramo, Stevens, et al. 2018, indicates a mean of 6% and, for two eggs collected near our study sites in Cadiz province, 6% and 8%). Furthermore, Gómez, Ramo, Stevens, et al.'s (2018) analysis of the UV and human‐visible‐spectrum reflectance of Kentish plover eggs indicates that UV and visible reflectance are highly correlated. For the egg basal, coloration *r* is 0.90 and for the spots 0.84 (using log‐transformed values; table 1 in Gómez, Ramo, Stevens, et al. 2018). That is, most of the variation between eggs is in lightness and spot patterning (Gómez, Ramo, Stevens, et al. 2018).

### 2.4 Pattern Analysis

In computer vision and perceptual psychology, there are well‐established methods for quantifying what, in those fields, is called visual texture. These involve decomposing a pattern into the information at different spatial scales and orientations. As in Michalis et al. (2017), Talas et al. (2017), Barnett, Cuthill, and Scott‐Samuel (2018) and Barnett, Michalis, et al. (2018) texture was characterised by the output of a log‐Gabor filter bank of six spatial scales and eight orientations, applied to the luminance plane of each image. The luminance signal was used as this is the main contributor to pattern vision for the species modelled (Jones and Osorio 2004; Kelber et al. 2003). In practice, the same texture analysis was used for all three visual systems (avian, carnivore, humans) because the calculated luminance values for the images (eggs and backgrounds) were so highly correlated: avian‐carnivore *r* = 0.96, avian‐human *r* = 0.99, human‐carnivore *r* = 0.98 (*n* = 2640; 30 samples from each of 88 nests). Gabor filters are a standard tool in image processing, used to describe pattern or detect visual structure. A single Gabor filter is a 2D sine or cosine wave of a given spatial frequency and orientation multiplied by a 2D Gaussian (normal) distribution of a given standard deviation. So, when multiplied by a patch of an image of the same size, the product will have the highest value when the pattern in the image‐patch matches the structure of the Gabor filter. When you multiply a whole image by a given Gabor filter (by successively moving it, pixel‐by‐pixel, across all the rows and columns of the image), the largest values will show you the areas of the image where the orientation and spatial frequency (from coarse to fine grain) match that Gabor. If you repeat this process with a whole set of Gabor filters of different spatial frequencies and orientations, you get a comprehensive description of the patterns of light and dark, of given sizes and orientations, in that image. Rather than, for example, describing a 256 × 256 pixel image with 65,536 numbers (the intensity values of the 65,536 pixels), the pattern in the image is described with 48 numbers (6 spatial scales and 8 orientations). Numerous vision specialists in the present era have concluded that the frequency and orientation representations exhibited by Gabor filters bear resemblance to the response properties of certain neurones in the human visual system (e.g., Field 1987; Field and Olshausen 1996; Olshausen and Field 1996; Ruderman et al. 1998). The log of the Gabor output (hence ‘log‐Gabor filter’) was used for statistical reasons: the output of a simple Gabor filter is always zero or positive, so correlated with mean luminance, while logged values can be negative.

**FIGURE 2:**
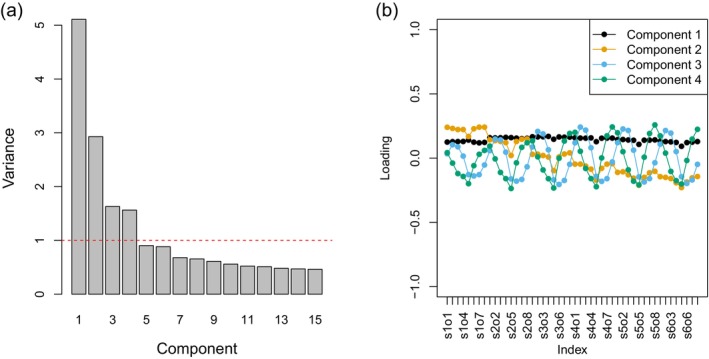
(a) The eigenvalues (variance) of the first 15 (of 48) principal components derived from Principal Component Analysis of the 48 log‐Gabor filter outputs used to describe the texture (spatial pattern) of the eggs and backgrounds. (b) The loadings of the principal components on the original 48 variables: That is, the contribution of the original variables to each component. The original Gabor variables (*x*‐axis labelled as Index) are named such that the first two letters (s1, s2, …, s6) describe the spatial scale (1 is fine detail, 6 is the coarsest detail) and the second two letters (o1, o2, …, o8) describe the orientation of the filter, from horizontal (o1) turning anti‐clockwise.

The output of log‐Gabor filters like this, when applied to natural images, is correlated because of the fractal nature (self‐similarity) of natural scenes (Burton and Moorhead 1987; Párraga et al. 2002; Turiel et al. 2000); for example, when there is high contrast at large spatial scales there tends to be high contrast at low spatial scales. Because of this, and to reduce the number of response variables to be analysed, Principal Component Analysis was carried out on the correlation matrix of the dataset using the *princomp* function in base R (the approach taken by Talas et al. 2017). Four components had eigenvalues greater than 1 (i.e., explained more variation than any of the original variables) and captured 83% of the total variation in the 48 Gabor outputs (54%, 18%, 6% and 5% for PC1 to 4, respectively). We can understand what the components represent by examining their loadings: the contribution of the original variables to each component (Figure 2). The first component (PC1) loads positively on all 48 original Gabor measures: it captures ‘contrast’ regardless of spatial scale (an image with a high value of PC1 would have areas of very light and very dark at both fine and coarse grain). PC2 loads positively on the Gabor filters capturing fine detail and negatively on those capturing coarse detail; an image with a high positive value of PC1 would have more fine detail and few large objects (e.g., sand), while a high negative value would indicate mainly large objects (e.g., pebbles). PC3 and 4 together capture the orientation of edges in an image. The fact that they appear as 180 out‐of‐phase sine waves in Figure 2b is really an artefact of the method: by definition, principal components must be orthogonal (uncorrelated), so, if PC3 captures the relative importance of one set of orientations, PC4 is always likely to show the opposite pattern of loadings. The specific angles involved are not of interest for this study (these eggs and backgrounds do not have patterns that have a strong directional component: they are not striped), unlike the orientation of striped moths on striped tree bark (Kang et al. 2012, 2013, 2014).

We expect that initial detection is likely to be from a distance where the patterning on an egg cannot be resolved. For example, a human with excellent vision in bright light can resolve 73 cycles per degree (Land and Nilsson 2012, table 3.1), so a black 1 mm line on a white background might be resolvable at 4.2 m. The pattern on plover eggs is of high contrast, but of lower contrast than black on white, and a typical avian egg predator such as a corvid has an acuity about half that of humans (ca. 30 cpd; e.g., magpie *Pica pica* , jackdaw *Coloeus monedula* or rook *Corvus frugilegus* ; table S1 in Caves et al. 2024), so the highest contrast fine detail might only be seen at under 1.7 m. For a red fox ( *Vulpes vulpes* ), with an estimated acuity of 8.7 cpd (Malkemper and Peichl 2018), the equivalent distance would be 0.5 m. So the average egg colour will be the primary determinant of initial visual detectability (mammals may use olfaction), but, once close‐up, patterning may become important. As the previous rough calculations suggest, how close is ‘close’ is highly variable across species, and we lack data on detection distances.

Note that the analysis is at the level of ‘egg‐sized’ objects and so any spatial variation in light and dark of greater surface area than an egg is not captured by our Gabor features or the principal components derived from them. From the predator's perspective, what is required is a sufficient contrast to favour closer inspection, even if initially the egg is not recognised as an egg. Nevertheless, it is the average coloration of an egg which will determine the maximum possible contrast between an egg, or clutch of eggs and the local background at a distance. The contrast between the whole clutch and local background will be lower than that of an individual egg and the background because the average of a clutch where individual eggs cannot be resolved will necessarily include some of the background (particularly so, if background materials are added to the nest by the parent; e.g., Stevens et al. 2017; Gómez, Ramo, Stevens, et al. 2018).

### 2.5 Statistical Analysis

Analysis of the differences in the means of the response variables describing colour and texture was by linear mixed models, using the function lmer from the lme4 package (Bates et al. 2015). With multiple replicates (3 eggs, 27 background samples) taken from each photograph/nest, ‘photograph’ was included as a random effect in all models. The predictors were either location (beach vs. salt marsh) or object type (egg vs. background) according to the question involved. The lmerTest package was used to obtain *p* values from *t*‐tests using Satterthwaite's method to calculate the appropriate degrees of freedom (Kuznetsova et al. 2017). For the texture measures based on PCA of the log‐Gabor filter outputs, we also carried out Multivariate Analysis of Variance (MANOVA) on all four principal components as joint response variables, using Pillai's trace as both a test statistic and measure of effect size equivalent to *R* ^2^ (Pillai 1955). This is a multivariate equivalent of *R* ^2^ in regression and is also equivalent to the partial *η* ^2^ statistic provided by the widely used statistics package SPSS. To obtain a *p* value, we used the approximate *F* test provided by the MANOVA function in R, based on Pillai's trace and the ratio of the location variance to the between‐nest (i.e., photograph) variance. The reason to use MANOVA to analyse the texture measures jointly was both as a protection against the elevated Type I (false positive) error rates of testing PC1 to PC4 separately, but also, and more importantly, because these statistical measures of texture do not have the same perceptual interpretation as the colour measures. That is, the neural processing of luminance and colour (hue) are to some degree understood, but perceptual dimensions of texture (‘pattern’) are not (Stoddard and Osorio 2019). Therefore, analysing all texture measures together seems a sensible conservative approach.

Of greater relevance to camouflage than differences between the mean colours or patterns of eggs and their backgrounds is their discriminability/confusability (Barnett et al. 2020, 2021; Michalis et al. 2017). If one imagines egg colours and background colours as two clouds of points in a colour space, how much do the distributions overlap (Endler and Mielke 2005)? This is a signal detection problem, with the egg features being the signal and the background features the noise (Merilaita et al. 2017). Unless the two distributions do not overlap, there is no single criterion that can perfectly distinguish one from the other: a threshold that classifies more eggs correctly will incorrectly classify more background features as belonging to eggs (false positives), and a threshold that classifies more backgrounds correctly will incorrectly classify more egg features as being part of the background (failed detections). That trade‐off can be visualised as an receiver operating characteristic (ROC) curve, where ‘sensitivity’ (correct classifications of eggs as eggs) is plotted against ‘specificity’ (correct classification of backgrounds as backgrounds) (Wickens 2002). This is standard practice in machine learning, as is the procedure of ‘cross‐validation’ of the discrimination model (Lantz 2013). The goodness of fit of a model to a set of data will always be better than the success of that model when applied to new data of the same type, known as ‘over‐fitting’. This is because some of the variation in any one sample of data is random noise, so a model fitted to those data will not fit a new sample (with different random sampling variation) as well as it did the original data. In machine learning, the solution to this is to fit a model to one set of data (‘training’) but test it with a different set of data (Lantz 2013). There are different ways you can do this (e.g., randomly divide the dataset in two, an approach taken by Barnett et al. (2021), when analysing camouflage in leaf‐mimicking toads), but we used the computationally more intensive leave‐one‐out cross‐validation, because it makes fuller use of the data (Lantz 2013). As the name suggests, the discrimination model is fitted to the data of all‐but‐one nest, then the model's success in correctly classifying the eggs and background samples from the remaining nest is assessed (the probability of the object being an egg, or background, is the output). The process is repeated for every nest, each time training the model on the other nests. The discrimination model used was a generalised linear mixed model with binomial error, fitted using the glmer function from the lme4 package. The response variable was object type (which could be an egg or background sample), the fixed effect predictors the colour or pattern metrics for different visual systems, and the random effect ‘photograph’ (each photograph—one per nest—provided three egg and 27 egg‐shaped background samples, so these 30 samples shared, among other things, the precise illumination at the time of taking the photograph). Classification errors were calculated using the confusionMatrix function from the caret package (Kuhn 2008), and ROC curves were fitted using the pROC function from the package of the same name (Robin et al. 2011).

## 3 Results

**FIGURE 3:**
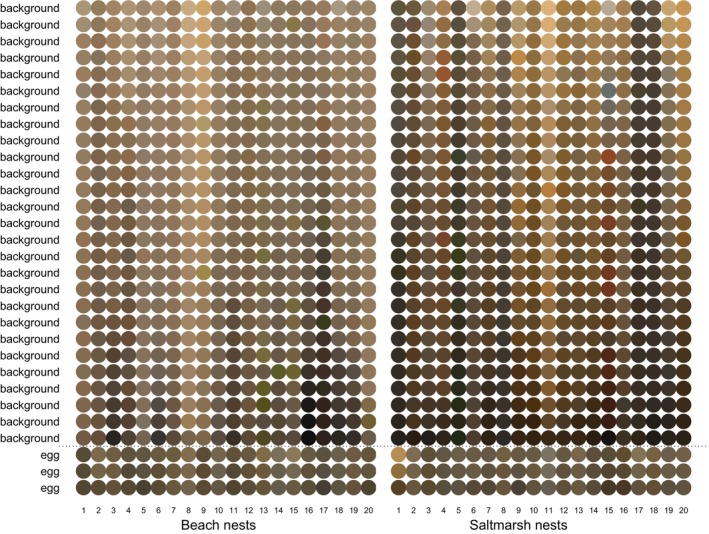
Examples of the colours of the three eggs in each nest and their respective 27 background samples based on a random sample of 20 nests from the beach and salt marsh. To aid visual comparison across habitats and between eggs and backgrounds, eggs and (separately) backgrounds have been ordered by luminance within each nest (= column).

Viewing the colours of eggs and backgrounds from the beach and salt marsh as they appear to us gives an immediate impression that the egg colours from both habitats are similar but, as a background, the salt marsh is more variable and, on average, darker than the beach (Figure 3). Statistical analysis, using linear mixed models, of the components of both avian and carnivore colour models mirrors this subjective view from human perception. Analysing all the data together with respect to both object type (egg/background) and location (beach/salt marsh), there are significant type × location interactions for all measures of colour and texture (Table 1). One can break this interaction down in two ways, and both are of interest. First, analysing eggs and backgrounds separately, do egg colours and textures differ between the beach and salt marsh (indicating either local adaptation or background choice), and the matching question of do the backgrounds differ? But another split of the data is of direct relevance to quantifying camouflage in the two habitats: analysing beach and salt marsh habitats separately, how do egg and background colours and textures differ and how confusable are they?

**TABLE 1:** Statistical tests of the interaction between object type (egg/background) and location (beach/salt marsh) for each of the colour and texture metrics, based on linear mixed models.

	*t* or *F*	df	*p*
Colour metrics			
Avian luminance	4.999	2550	< 0.001
Avian RG	4.507	2550	< 0.001
Avian YB	11.706	2550	< 0.001
Carnivore luminance	7.841	2550	< 0.001
Carnivore YB	11.504	2550	< 0.001
Texture metrics			
PC1‐4 jointly (*F*)	33.910	4, 2547	< 0.001
PC1	2.234	2550	0.026
PC2	5.801	2550	< 0.001
PC3	6.944	2550	< 0.001
PC4	6.624	2550	< 0.001

*Note:* The dependent variables describing ‘colour’ are separated into an achromatic component (luminance) and chromatic opponent channels, two (red‐green and blue‐yellow) for an avian predator and one (blue‐yellow) for a mammalian predator. Visual texture (‘pattern’) is described by four principal components derived from an original set of 48 log‐Gabor filters. The test statistic for all measures is Satterthwaite's *t* from linear mixed models, except for the joint analysis of the texture measures PC1 to 4, which is an approximate *F*‐test from MANOVA.

First, do the eggs in the two habitats differ in appearance? To an avian predator, the beach and salt marsh eggs are not significantly different in mean luminance, red‐green or yellow‐blue colour measures (Table 2). Similarly, to a mammalian carnivore, there is no detectable difference in mean luminance or yellow‐blue colour measures (Table 2). There is also no detectable difference in texture measures of egg patterning (Table 2), assumed to be similar for both visual systems. Conversely, to an avian predator, the salt marsh backgrounds are on average darker and slightly, but significantly, more reddish‐brown than the beach (higher red‐green and yellow‐blue values; Table 2). Likewise, the modelling indicates that a mammalian carnivore would perceive the salt marsh backgrounds as, on average, darker and browner (Table 2). The textures of the backgrounds also differ, the salt marsh having higher overall contrast (PC1), being coarser‐grained and having a different distribution of edge orientations from the beach (PC3 and 4; Table 2). This corresponds with the higher proportion of pebbles and patches of vegetation in the salt marsh.

**TABLE 2:** Comparison of mean colour and texture measures between the beach and salt marsh habitats, separately for eggs and backgrounds.

Measure	Eggs			Backgrounds		
	*t* or *F*	df	*p*	*t* or *F*	df	*p*
Colour metrics						
Avian luminance	1.821	86	0.072	5.497	86	< 0.001
Avian RG	0.100	86	0.920	2.335	86	0.022
Avian YB	0.431	86	0.667	4.872	86	< 0.001
Carnivore luminance	1.661	86	0.100	8.706	86	< 0.001
Carnivore YB	0.572	86	0.569	4.837	86	< 0.001
Texture metrics						
PC1‐4 jointly (*F*)	1.295	4,83	0.279	30.234	4,83	< 0.001
PC1	1.408	86	0.163	3.975	86	< 0.001
PC2	2.167	86	0.033	9.155	86	< 0.001
PC3	1.708	86	0.091	2.852	86	0.005
PC4	1.359	86	0.178	3.033	86	0.003

*Note:* The dependent variables describing ‘colour’ are separated into an achromatic component (luminance) and chromatic opponent channels, two (red‐green and blue‐yellow) for an avian predator and one (blue‐yellow) for a mammalian predator. Visual texture (‘pattern’) is described by four principal components derived from an original set of 48 log‐Gabor filters. The test statistic for all measures is Satterthwaite's *t* from linear mixed models, except for the joint analysis of the texture measures PC1 to 4, which is an approximate *F*‐test from MANOVA.

**FIGURE 4:**
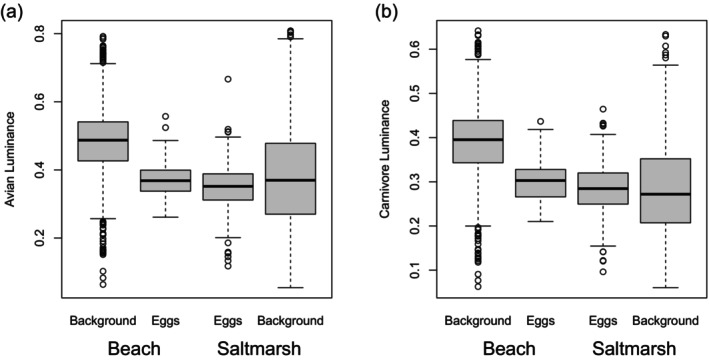
(a) Boxplots of the avian luminance (double cone catch) distribution of background samples and eggs from the beach and salt marsh habitats. (b) The equivalent for a mammalian carnivore, based on ferret vision. The thick horizontal line is the median; the box spans the lower to upper quartile; the ‘whiskers’ extend to the last data point within 1.5 inter‐quartile ranges of the nearest quartile; the open circles are points outside the whiskers.

**FIGURE 5:**
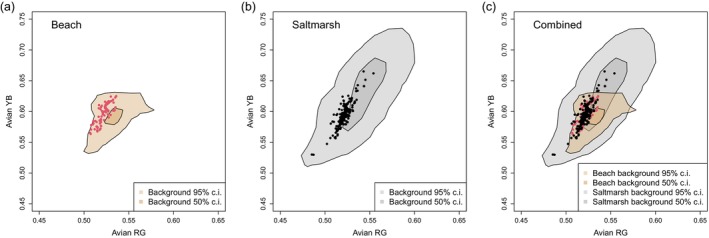
Avian‐perceived colours of eggs and backgrounds in (a) the beach and (b) salt marsh habitats. (c) The two datasets plotted together to show regions of overlap in egg and background distributions. The two axes describing colour are a yellow‐blue (YB) and a red‐green (RG) opponent channel. The background colour distributions in each panel are summarised by their 95% and 50% kernels, with the hues of eggs superimposed as individual points.

In terms of the mean values of colour and texture metrics, although for carnivore vision there is no significant difference in egg and background luminance for the salt marsh samples, broadly speaking, plover eggs do not match the background in either habitat, for either avian or carnivore visual system (Table 3). The significant object‐type*location interaction reported earlier (Table 1) arises because the eggs mismatch the two habitats in different ways. In both habitats, for avian vision eggs are on average darker than the background, but in the beach habitat, the difference is larger (Table 3; Figure 4). On the beach, the eggs are slightly yellower than the background, but in the salt marsh, less yellow; the eggs are less red than the backgrounds in both habitats but the difference is greater in the salt marsh (Table 3; Figure 5). Note that ‘yellower’ and ‘less red’ are purely with respect to these colour dimensions; as mentioned earlier (Section 2.3), these are all shades of brown.

**TABLE 3:** Comparison of mean colour and texture measures between eggs and backgrounds, separately analysed for the beach and salt marsh habitats.

	Beach				Salt marsh			
	Effect	*t* or *F*	df	*p*	Effect	*t* or *F*	df	*p*
Colour metrics								
Avian luminance	−0.105	10.910	811	< 0.001	−0.030	3.208	1739	0.001
Avian RG	−0.011	9.847	811	< 0.001	−0.020	16.650	1739	< 0.001
Avian YB	0.004	2.380	811	0.018	−0.034	16.390	1739	< 0.001
Carnivore luminance	−0.086	10.960	811	< 0.001	0.004	0.533	1739	0.594
Carnivore YB	0.006	3.073	811	0.002	−0.038	15.510	1739	< 0.001
Texture metrics								
PC1‐4 jointly (*F*)	0.453	167.100	4808	< 0.001	0.456	363.890	41,736	< 0.001
PC1	1.807	4.405	811	< 0.001	0.481	1.346	1739	0.1785
PC2	3.672	15.103	811	< 0.001	5.228	36.310	1739	< 0.001
PC3	1.878	11.776	811	< 0.001	0.598	5.894	1739	< 0.001
PC4	1.919	12.283	811	< 0.001	0.718	7.179	1739	< 0.001

*Note:* With the exception of the joint analysis of the texture measures PC1 to PC4, where the effect measure is Pillai's trace from MANOVA, the effect size is the difference in standardised mean between egg and background (so a negative sign means a lower value for the egg than the background). The test statistic for all measures is Satterthwaite's *t* from linear mixed models, except for the joint analysis of the texture measures PC1 to 4, which is an approximate *F*‐test from MANOVA.

Considering texture (Table 3; Figure 7), the mismatch between eggs and backgrounds for PC1 (overall contrast) is greater in the beach than salt marsh, the latter habitat having a greater range of dark and light objects than the beach and the eggs, being essentially two‐tone (dark maculation on lighter background), also high in contrast. Conversely, the mismatch for PC2 (relative amount of fine‐ to coarse‐grain patterning) is greater for the salt marsh, the latter having more pebbles that are smaller than an egg but larger in size than the finer‐grained maculation of the eggs. PC3 and PC4 are much more similar between the habitats, but the means are still significantly different (Table 2) and the eggs differ more from the beach means than the salt marsh (Table 3; Figure 7).

**FIGURE 6:**
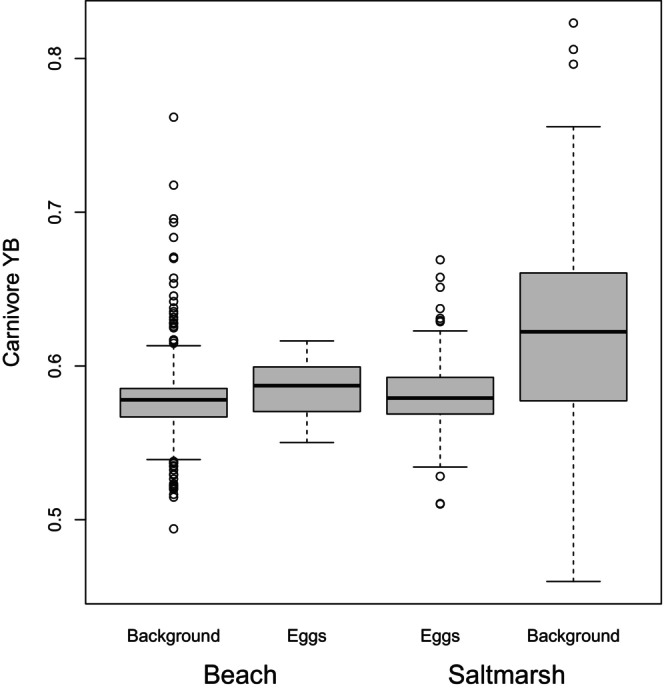
Boxplots of the modelled carnivore perceived hues of background samples and eggs from the beach and salt marsh habitats. Carnivores are dichromats, so the colours of samples are represented in a one‐dimensional yellow‐blue (YB) opponent colour space. The thick horizontal line is the median; the box spans the lower to upper quartile; the ‘whiskers’ extend to the last data point within 1.5 inter‐quartile ranges of the nearest quartile; the open circles are points outside the whiskers.

**FIGURE 7:**
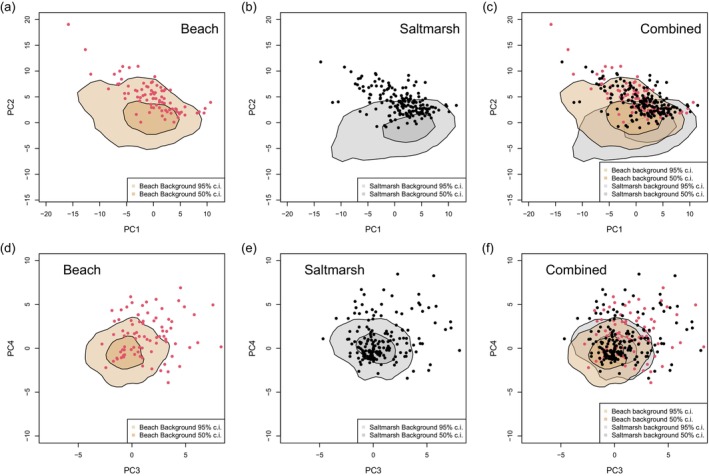
Differences between backgrounds and eggs for the principal components describing pattern (texture) (top row: PC2 vs. PC1; bottom row: PC4 vs. PC3) for beach (a, d) and salt marsh (b, e) habitats. Background samples are summarised by their 95% and 50% kernels, with the values for eggs superimposed as individual points. Panel (c) combines the data from (a) and (b) to show regions of overlap in egg and background distributions. Panel (f) is the equivalent graphs for PC4 plotted against PC3. PC1 captures ‘contrast’ regardless of spatial scale; PC2 measures the relative amount of fine to coarse detail; both PC3 and 4 capture variation in line and edge orientations.

Up to now, we have considered egg‐background differences. Figures 5c and 7c,f facilitate the comparison of habitats, a way that is relevant to understanding whether the eggs have ‘compromise camouflage’ intermediate to the beach and salt marsh backgrounds. The hues of the beach and salt marsh backgrounds, as seen by birds (Figure 5) or mammalian carnivores (Figure 6), while on average different (Table 2), overlap considerably. The eggs' hues fall in the regions of overlap, although the egg luminance, for either visual system, is closer to the salt marsh in distribution (Figure 4). The patterning of the eggs, characterised by overall contrast (PC1) and the relative amount of fine‐grained detail (PC2), shows greater overlap with the distribution observed for the beach background. However, egg patterning, in terms of contrast (PC1) and relative amount of fine to coarse detail (PC2), does not align closely with the central tendency of either background distribution (Figure 7a,b). Principal components 3 and 4 are more similar between the two habitat types (Figure 7d–f).

Although eggs and backgrounds in either habitat differ in their mean values for all aspects of colour and texture, it is clear that the distributions overlap considerably for both avian and carnivore vision (Figures 4, 5, 6, 7). Confusion matrices and ROC curves based on classification models with leave‐one‐out cross‐validation quantify that overlap: the discriminability (converse: confusability) of the colours and textures. Classification accuracy appears high, but it is important to remember that there were nine times as many background as egg samples (27 background samples and 3 eggs per nest), so the naïve rule of ‘classify all objects as background’ would have an accuracy of 0.9 (all backgrounds correct, all eggs incorrect). This is known as the ‘no information rate’ and the obtained accuracies using colour measures alone (for human, bird or carnivore vision) are lower than this. Few or no eggs are correctly classified based on colour alone. There is no clear difference between the habitats (correct identification of eggs is slightly higher in the salt marsh, but correct identification of backgrounds is slightly higher in the beach; Table 4). Incorporating texture information yields greater classification success, with pattern alone being a significantly better criterion than the naïve rule, with around 70% of eggs correctly classified (vs 0 using the naïve rule). Using both colour and texture information is better still, with human vision achieving perfect classification and outperforming that of birds, and both outperforming carnivore vision. In the salt marsh habitat, the classification success of carnivore vision drops to that of the naïve rule; with hardly any eggs classified correctly, but 98% of backgrounds correctly classified, the model is essentially matching the no‐information rule of classifying all objects as backgrounds.

**TABLE 4:** Measures of classification success based on binomial mixed models with leave‐one‐out cross‐validation. Separate analyses for beach and salt marsh habitats.

Measure	Accuracy				Sensitivity		Specificity	
	Beach	*p*	Salt marsh	*p*	Beach	Salt marsh	Beach	Salt marsh
Human colour	0.86	1.00	0.85	1.00	0.00	0.10	0.96	0.94
Avian colour	0.87	1.00	0.84	1.00	0.00	0.02	0.97	0.93
Carnivore colour	0.88	0.98	0.88	0.99	0.00	0.01	0.98	0.98
Pattern	0.94	< 0.001	0.95	< 0.001	0.71	0.69	0.96	0.98
Human both	0.98	< 0.001	1.00	< 0.001	0.90	1.00	0.98	1.00
Avian both	0.97	< 0.001	0.94	< 0.001	0.88	0.86	0.98	0.95
Carnivore both	0.96	< 0.001	0.88	0.99	0.80	0.01	0.98	0.98

*Note:* Accuracy is the proportion of eggs and backgrounds classified correctly. The *p value* is the probability that the accuracy is better than the no‐information rate of 0.9. Sensitivity is the proportion of eggs correctly classified as eggs, Specificity is the proportion of background samples correctly classified as backgrounds.

**FIGURE 8:**
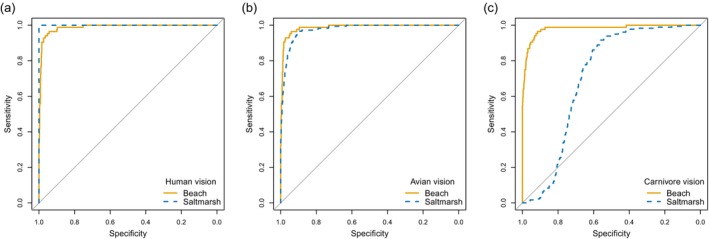
Receiver operating characteristic (ROC) curves for (a) humans, (b) avian and (c) mammalian carnivore predators, using both colour and texture information, separately plotted for beach (orange solid lines) and salt marsh (blue dashed lines). Sensitivity is the proportion of eggs correctly classified as eggs; specificity is the proportion of background samples correctly classified as backgrounds.

**FIGURE 9:**
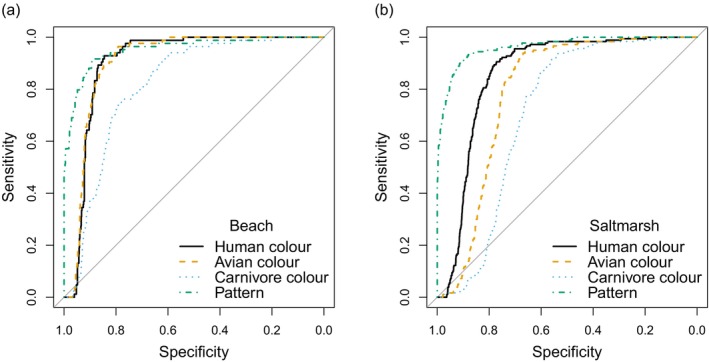
Receiver operating characteristic (ROC) curves using colour information only (human, avian and mammalian carnivore vision plotted separately), and texture for (a) beach and (b) salt marsh habitats. Sensitivity is the proportion of eggs correctly classified as eggs; specificity is the proportion of background samples correctly classified as backgrounds.

The classification accuracy discussed above assumes that the costs of failing to identify an egg correctly (Type II error: failed detection) is the same as failing to identify a background sample correctly (Type I error; false positive), which may not be true. Therefore, it is helpful to look at the complete trade‐off between type I and II errors as shown in ROC curves (Figures 8 and 9). Using both colour and texture information, as would be the case for a predator very close to the nest, the modelling predicts no trade‐off for humans searching on a salt marsh (no errors of either kind) and only a small trade‐off on the beach (Figure 8 and Table 4). However, although classification performance for avian vision is also very high, an avian predator seeking to detect all eggs (sensitivity = 1) would have to accept a false positive rate over 20% (specificity < 0.8) in either habitat (Figure 8b). Carnivore classification performance on the salt marsh is predicted to be poor: to achieve 100% egg detection, a false positive rate over 75% (specificity < 0.25) would be incurred (Figure 8c).

The relative contributions of colour and texture can be separated by calculating the ROC curves for each type of information (Figure 9). The role of colour alone is relevant for detection at a distance, that is, at distances above which the patterns on the eggs and background sample of a similar size cannot be resolved. Using texture information alone, high sensitivity can be achieved at higher specificities than using colour alone in either habitat for any of the three visual systems. Therefore, it is the texture that is the major contributor to the classification success seen in Figure 8, using all available information. On the beach, humans and birds face similar trade‐offs when using colour alone, with a much steeper trade‐off (the false positive rate for a given level of egg detection) for mammalian carnivore vision. On the salt marsh, the strength of trade‐off is human < avian < carnivore and somewhat steeper than on the beach.

## 4 Discussion

For an avian (or human) predator that is close enough to nests to see the surface patterning, plover eggs are easily distinguishable from the background in either the beach or salt marsh habitats. Even without formal analysis showing noticeably higher values of PC2 (high spatial frequencies) in the eggs than backgrounds (PC2, the *y*‐axis in Figure 7a,b), this could be anticipated because the egg patterns consist of small dark dots and wispy lines, unlike common small background objects like pebbles and bits of vegetation. Conversely, at a distance beyond which egg patterns cannot be resolved and only average colour can be used by predators, the eggs are quite cryptic. While there is a statistically significant difference between the means of all colour metrics, for each visual system investigated, the distributions overlap considerably and so egg and background colours are readily confused. Because egg colours are less variable than background colours, there are a large number of background objects which could never be eggs (based on colour), but there are still a considerable number that could be. In technical terms, predator can set a colour‐based threshold for classifying an object as ‘egg’ or ‘background’, but a high detection rate for eggs can only be achieved at the expense of a high false positive rate (backgrounds misclassified as eggs). The classification performance for modelled mammalian carnivore vision is markedly worse than bird or human, particularly in the salt marsh habitat. This does not mean that terrestrial carnivores are necessarily less of a threat to nesting success for these plovers; smell is likely to be important for nest detection by mammals and predation may also occur at night (Ellis et al. 2018; Grieves et al. 2022; Li et al. 2023).

There are multiple reasons why we should be wary of generalising our results: the sample size, particularly for the beach (*N* = 28), was modest and the data come from one breeding season in one geographical location, whereas the Kentish plover has a very broad distribution, spanning Africa, Europe and Asia (Meininger et al. 2009). Our photographs were taken in conditions of sun and clear skies, and these were the viewing conditions that predominated in the breeding season. However, changes in cloud cover and thus the ratio of direct to diffuse illumination will change contrast and colour tone in the scene as a whole (Endler 1993; Lovell et al. 2005; Penacchio et al. 2015; Szala et al. 2023). The sun's angle, as affected by the time of year and day, will also affect the intensity and directionality of shadows in a scene, and Szala et al. (2023) showed that it affects measures of coloration from birds' eggs. That said, measurements were still repeatable across lighting conditions in their study and, in ours, there were no consistent differences in times of photography between the two habitat types. Nevertheless, all the above factors add noise to the data. It must also be emphasised that this study only investigated background matching: whether the surface colours and patterning of eggs were similar to those of the background (Merilaita and Stevens 2011). No aspects of egg shape were considered. Depending on the height of the sun and surrounding objects, the cast shadow on the adjacent ground or the shape‐from‐shading cues created by directional illumination could reveal an egg (Cott 1940; Penacchio et al. 2015). This is likely to be a greater problem in an open habitat like the beach, where vegetation does not create shading or its own cast shadows. Eggs are geometrically very regular objects, unlike stones of a similar size, and so the outline itself could be a cue to predators (Cott 1940; Webster et al. 2015). This may be relevant to understanding the maculation on the eggs which, at face value, does not aid background matching when a predator is close. Previous studies have shown that eggs exhibiting a higher number of spots possess improved camouflage (Gómez et al. 2016; Montevecchi 1976; Troscianko, Wilson‐Aggarwal, Spottiswoode, and Stevens 2016; Troscianko, Wilson‐Aggarwal, Stevens, and Spottiswoode 2016). However, as opposed to matching the background, the dark maculation could act as edge‐disruptive coloration, suggested by Lovell et al. (2013) to be a likely factor in the camouflage of quail eggs, or surface‐disruptive coloration to reduce the homogeneity of colour that might itself be revealing (Stevens et al. 2009). That said, if contrasting colour patches are there to create disruptive camouflage, why are they not larger? Larger colour patches would be more effective at edge‐disruption because the true edge would be interrupted more and false edges would be more distinctive (Espinosa and Cuthill 2014; Stevens and Cuthill 2006). Also, disruptive coloration is most effective when combined with background matching (Fraser et al. 2007; Stevens et al. 2006). All this points to disruption being an unlikely function of the maculation on this species' eggs. Other possibilities are that larger dark patches, because of heat absorption, are too costly in terms of thermoregulation when a parent is off the nest (Gómez et al. 2016; Wisocki et al. 2020). Having a mixture of lighter base colour and small maculation patches may achieve an average colour that is a good match to the background, but at lower cost than a darker, evenly coloured egg. This is speculation, but the combined thermal and visual consequences of different patterns of maculation (as opposed to average colour) should be investigated. The final possibility is that there is some other habitat, not investigated, to which the birds are better adapted, and that the sites in our study were peripheral, suboptimal, choices. This seems unlikely because other accounts of Kentish plover breeding ecology, across the world, discuss habitats very similar to the ones in this study (AlRashidi et al. 2010, 2011; Kosztolányi et al. 2003; Kosztolanyi et al. 2007; McDonald et al. 2022; Székely and Cuthill 1999).

Although the two habitats studied, beach and salt marsh, were superficially quite visually different (the former dominated by sand, the latter with muddy and vegetated areas), there was considerable overlap in both colour and pattern: both contained sandy and pebbly areas. Furthermore, and importantly, it was the overlapping areas (in terms of colour and pattern) which the plover eggs matched. Therefore, although based on overall subjective appearance of the habitats, we had predicted that the birds' eggs might display local adaptation, either diverging in egg appearance in the two habitats to matching one habitat or the other, or to show intermediate (compromise) camouflage, the birds did none of these things. Our interpretation is that they specialised on the microhabitat characteristics which allowed successful camouflage (from a distance) in both habitats. This hypothesis needs to be tested independently, ideally with a preference experiment, because there remains the possibility that poorly camouflaged nests had already been lost to predation before we could locate them.

Stevens et al. (2017) presented two mechanisms to explain how birds might achieve nest camouflage. The first is adaptation through natural selection, whereby the better camouflaged eggs are the ones that survive predation and so these characteristics are transmitted and refined over successive generations. The second, and not mutually exclusive, hypothesis suggests that birds learn to recognise their own eggs over time, or there is some genetic correlation between egg appearance and nest‐site‐preference, enabling them to make informed decisions regarding their placement. Both processes are credible, as there are correlations between both egg coloration and patterning with nesting habit (Kilner 2006) and there is supporting evidence from studies of habitat choice (Gómez, Ramo, Troscianko, et al. 2018; Stevens et al. 2017). The extent to which this is true in our study system was not directly investigated. Furthermore, no aspects of the appearance of the nest as a whole and the extent to which parents alter its appearance were investigated. Effective camouflage will often be determined by behaviour in addition to surface pigmentation (Stevens and Ruxton 2019). For instance, both Stevens et al. (2017) and Gómez, Ramo, Troscianko, et al. (2018) showed that individual plovers (and some other ground‐nesting species) not only selected nesting locations to which their eggs were closely matched in predator‐specific colour measures, but also changed the visual environment by covering their nests with materials that blended in with the surroundings. Hancock et al. (2023) recently used 3D reconstruction of the terrain around Northern lapwing ( *Vanellus vanellus* ) nest to show that, despite nesting in the open, the parents exploited slight variations in elevation such that the eggs would not be visible to a terrestrial predator from even 1.5 m away.

A final general point should be emphasised from the approach taken in this study to studying camouflage. In natural environments, a predator not only has to cope with perceptual limitations such as spectral sensitivity, receptor noise and acuity (e.g., Caves et al. 2025), it has to discriminate between a target and a background each comprising multiple possible colours. The signal detection approach taken in our study is a useful way of quantifying the confusability of these distributions.

## Author Contributions

**Alexandra Grandón‐Ojeda:** conceptualization (supporting), data curation (supporting), formal analysis (supporting), funding acquisition (lead), investigation (lead), methodology (supporting), writing – original draft (lead). **Tamás Székely:** conceptualization (equal), funding acquisition (supporting), methodology (supporting), project administration (lead), resources (supporting), supervision (equal), writing – review and editing (equal). **Robert N. Kelsh:** conceptualization (supporting), project administration (supporting), supervision (equal), writing – review and editing (equal). **Alejandro Pérez‐Hurtado:** investigation (supporting), methodology (supporting), project administration (supporting), resources (lead), supervision (supporting), writing – review and editing (equal). **Innes C. Cuthill:** conceptualization (equal), data curation (lead), formal analysis (lead), investigation (supporting), methodology (lead), project administration (supporting), resources (supporting), supervision (equal), writing – original draft (supporting), writing – review and editing (lead).

## Conflicts of Interest

The authors declare no conflicts of interest.

## Data Availability

Data and R script are available at the University of Bristol data repository, data.bris, at https://doi.org/10.5523/bris.31ab42htfown02izy42gknlkcj.
